# A Handbook of Pathological Anatomy and Histology

**Published:** 1885-05

**Authors:** 


**A Handbook of Pathological Anatomy and Histology.** With an Introductory Section on Post Mortem Examinations and the Method of Preserving and Examining Diseased Tissues. By FRANCIS DELAFIELD, M. D., Professor of Pathology and Practical Medicine College of Physicians an Surgeons, New York, and T. MITCHELL PRUDDEN, M. D., Lecturer on Normal Histology, Director of the Pathological Laboratory. New York: Wm. Wood & Co., 56 Lafayette Place. 1885.

This admirable book supplies all the needs of students and practitioners who wish to add a knowledge of the lesions of disease to that of its clinical symptoms. The work comprises instructions in the methods of making post mortem examinations, of the preservation and preparation of diseased tissues for microscopical examination, of the lesions of all the different parts of the body, of the general diseases, of violent deaths, and of deaths from poisoning. The description of tumors and the account of such general processes as inflammation and degeneration are most excellent. The illustrations are abundant and unusually good. Indeed, the whole work deserves the most unqualified commendation.

